# Revealing the Na storage behavior of graphite anodes in low-concentration imidazole-based electrolytes†

**DOI:** 10.1039/d3sc06640a

**Published:** 2024-04-05

**Authors:** Wei Zhao, Chunting Wang, Zhenjie Cheng, Cheng Zheng, Qian Yao, Jun Pan, Xiaojian Ma, Jian Yang

**Affiliations:** a Key Laboratory of Colloid and Interface Chemistry Ministry of Education, School of Chemistry and Chemical Engineering, Shandong University Jinan 250100 P. R. China; b School of Physical and Mathematical Sciences, Nanyang Technological University Singapore 637371 Singapore jun.pan@ntu.edu.sg

## Abstract

The thermodynamic instability of Na^+^-intercalated compounds is an important factor limiting the application of graphite anodes in sodium-ion batteries. Although solvent co-intercalation is recognized as a simple and effective strategy, the challenge lies in the lack of durable electrolytes. Herein, we successfully apply low-concentration imidazole-based electrolytes to graphite anodes for sodium-ion batteries. Specifically, low concentrations ensure high ionic conductivity while saving on costs. Methylimidazole molecules can be co-intercalated with Na^+^, and a small amount of unreleased solvated Na^+^ serves the dual purpose of providing support to the graphite layer and preventing peeling off. The interphase formed in imidazole is more uniform and dense compared with that in ether electrolytes, which reduces side reactions and the risk of internal short circuits. The obtained battery demonstrates a long cycle life of 1800 cycles with a capacity retention of 84.6%. This success extends to other imidazole-based solvents such as 1-propylimidazole and 1-butylimidazole.

## Abstract 2

Low-concentration imidazole-based electrolytes were employed in graphite anodes for sodium-ion batteries *via* a co-intercalation mechanism. The resulting battery exhibits an impressive cycle life of 1800 cycles with a capacity retention of 84.6%.

## Graphical abstract

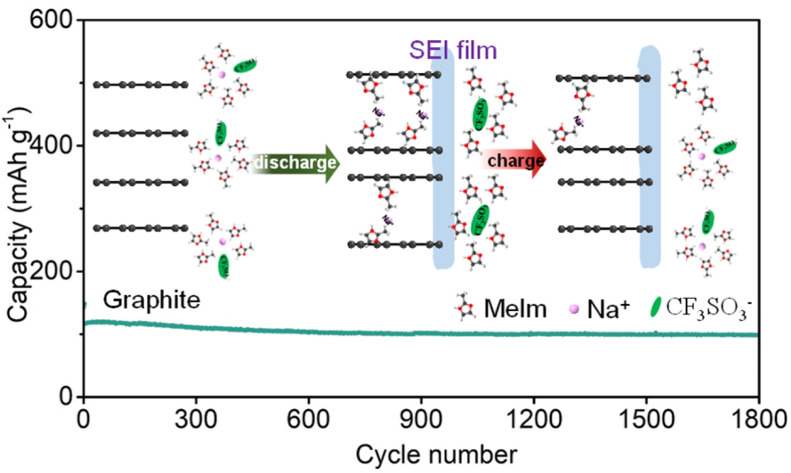

## Introduction

Sodium-ion batteries (SIBs) are emerging as a promising alternative to lithium-ion batteries (LIBs) in power and energy storage applications.^1–3^ The growing interest in SIBs stems from their similar production processes to LIBs, as well as their lower production costs.^4–6^ Graphite, renowned for its commendable conductivity and cost-effectiveness, stands out as a well-established commercial anode material for LIBs. However, its sodium storage capacity in conventional ester electrolytes is nearly negligible. This limitation is attributed to the thermodynamic instability of Na–graphite intercalation compounds (GICs).^7,8^

To mitigate the adverse interaction between Na and graphite, several strategies have been proposed, such as expanding the graphite interlayer and achieving the co-intercalation of Na^+^ and solvents. While expanded graphite demonstrates favorable sodium storage behavior, its synthesis process is complex, resulting in poor product consistency and environmental concerns.^9,10^ Another simple and effective method involves creating solvated sodium ion structures capable of intercalating between graphite layers. Currently, ether solvents are recognized molecules capable of intercalation in a solvated form.^11–13^ Jache *et al.* presented the co-intercalation phenomena of Na^+^ and ether-based solvents to overcome the mismatch of Na^+^ and graphite lattice distance.^14^ The co-intercalation is attributed to the strong solvation effect between sodium ions and ether molecules,^15^ as well as the nearly no solid electrolyte interphase (SEI) film formed by graphite in ether electrolytes.^16^ However, challenges, including material peeling and the risk of internal short circuits (due to an inability to charge to the set voltage), are encountered in the sodium storage of graphite in ether-based electrolytes.^17^ Therefore, there is an urgent need to develop an economical and stable electrolyte system to achieve efficient sodium storage in graphite materials.

Herein, a new low-concentration imidazole-based electrolyte is screened based on donor number (DN), dielectric constant, and ionic conductivity, which proves the cycle stability of graphite anodes in sodium-ion batteries. Firstly, the use of a low-concentration salt ensures high ionic conductivity while reducing the electrolyte cost. Secondly, the solvated Na^+^ ions, initially intercalated but partially unreleased, serve a dual purpose. They provide structural support to the graphite layer, facilitating the intercalation/deintercalation of solvated sodium ions in subsequent cycles, thereby improving rate performance. Additionally, these unreleased solvated-Na^+^ ions interact with 1-methylimidazole (Melm) molecules, hindering the peeling of the graphite layer and contributing to enhanced cycle stability. Thirdly, in comparison to ether electrolytes, the solid electrolyte interface (SEI) layer formed in Melm exhibits greater uniformity, diminishing side reactions and mitigating the risk of internal short circuits during cycling. Based on 0.2 M electrolyte, the obtained half-cell (C//Na) achieves a long life of over 1800 cycles (∼84.6% capacity retention) and the full cell (NaTi_2_(PO_4_)_3_//C) shows a long cycle life of over 2800 cycles. Furthermore, encouraging results are also obtained when extended to 1-propylimidazole (Prlm) and 1-butyimidazole (Bulm) solvents. These findings offer valuable insights for the selection of new electrolytes in sodium-ion battery applications.

## Results and discussion

### Electrolyte properties and electrochemical performance

**Fig. 1:**
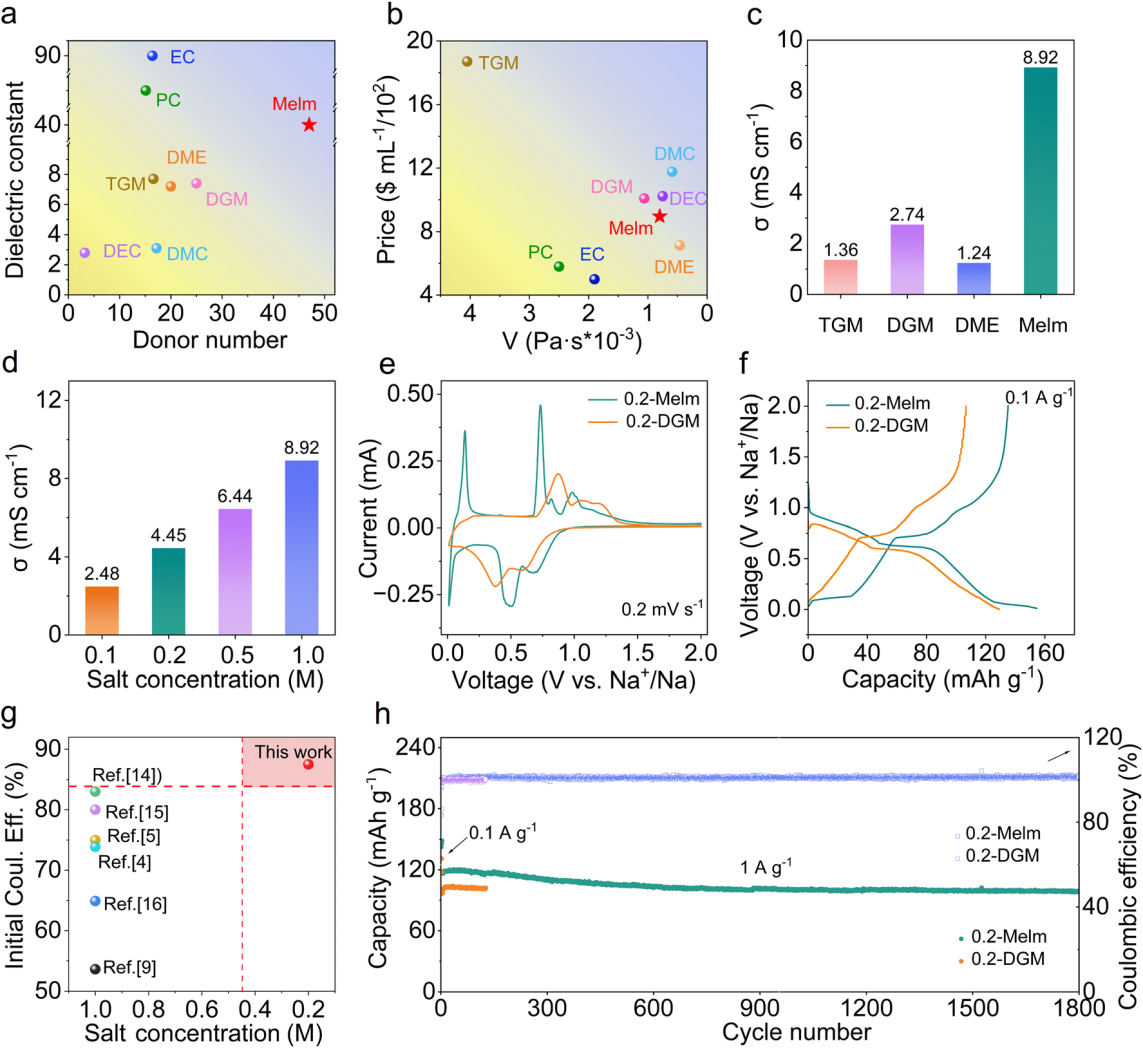
(a) DN *versus* dielectric constant plot of different solvents. (b) Viscosity *versus* price plot of different solvents. (c) Ionic conductivity of 1 M NaSO_3_CF_3_ in different solvents. (d) Ionic conductivity of NaCF_3_SO_3_ in Melm with different salt concentrations. (e) CV curves of MCMB anodes in 0.2-Melm and 0.2-DGM. (f) Galvanostatic voltage profiles of MCMB anodes in 0.2-Melm and 0.2-DGM at 0.1 A g^−1^. (g) Initial coulombic efficiency (ICE) comparison diagram. (h) Long-term cycling performances of MCMB electrodes in 0.2-Melm and 0.2-DGM.

To achieve co-intercalation of solvent and Na^+^, the solvent needs to be strongly coordinated with Na^+^.^18^ Two important parameters measuring solvation ability are DN and dielectric constant. Larger values indicate stronger solvation.^19^ Melm has a higher DN and higher dielectric constant, which can meet the requirements (Fig. 1a). In addition, Melm exhibits lower viscosity and a more affordable price than other solvents (Fig. 1b). The ionic conductivity exceeds that of traditional ether solvents at the same salt concentration (Fig. 1c). After comprehensive consideration, Melm emerges as the most suitable choice. Salt concentration crucially influences electrolyte cost, making it pivotal to achieve Melm application at low concentrations. Hence, three common sodium salts (NaPF_6_, NaCF_3_SO_3_, and NaClO_4_) were utilized to prepare the electrolytes (Table S1†) to identify the optimal electrolyte. The Raman spectrum revealed an abundance of free anions in 0.1 M NaCF_3_SO_3_ in Melm (0.1-Melm) and 0.2 M NaCF_3_SO_3_ in Melm (0.2-Melm) electrolytes (Fig. S1†) compared to other concentrations, suggesting sodium ions were surrounded by solvent molecules.^20^ This environment facilitates sodium ion intercalation into the graphite layer in a solvated form (Fig. S2†). Although the solvation structure in 0.2-Melm resembles that in 0.1-Melm, the ionic conductivity of 0.2-Melm (Fig. 1d) reaches 4.45 mS cm^−1^, twice that of 0.1-Melm (2.48 mS cm^−1^). Furthermore, 0.2-Melm exhibits higher capacity and superior cycle stability compared to 0.2 M NaPF_6_ in Melm and 0.2 M NaClO_4_ in Melm (Fig. S3 and S4†). Therefore, 0.2-Melm was chosen for subsequent comparative experiments and characterization.

Given that ethers are recognized solvents for solvation intercalation, diglyme (DGM) was selected as the reference for electrochemical performance (Fig. S5†). A comparative analysis reveals that 0.2-Melm exhibits lower viscosity, higher ionic conductivity, and superior solvation capability compared to 0.2-DGM (Fig. S6–S8†). Next, mesophase carbon microbeads (MCMB) were employed as the anode to assess electrochemical performance using 0.2-Melm and 0.2-DGM electrolytes (Fig. S9†). The redox peaks in the cyclic voltammetry (CV) curves represent the formation of different graphite interlayer compounds. Notably, the polarization voltage in 0.2-Melm is smaller than that in 0.2-DGM, indicating faster reaction kinetics in 0.2-Melm (Fig. 1e).^21^ In contrast to the ether electrolyte, MCMB in 0.2-Melm has an additional pair of redox peaks at 0.05/0.1 V, gradually disappearing with increasing current (Fig. S10†). This suggests an additional Na^+^ intercalation reaction at low voltages.^22^ The initial coulombic efficiency of MCMB in 0.2-Melm is 87.6% (Fig. 1f), surpassing that reported in several other articles (Fig. 1g).^15,23–27^ Moreover, MCMB in 0.2-Melm presents superior rate performance (Fig. S11†), demonstrating a capacity of 128.8 mA h g^−1^ at 0.5 A g^−1^, 123.6.5 mA h g^−1^ at 1 A g^−1^, and 117.1 mA h g^−1^ at 2 A g^−1^. Impressively, the MCMB anode in 0.2-Melm sustains a long-term cycle performance over 1800 cycles at a current density of 1 A g^−1^, maintaining a capacity of 98.8 mA h g^−1^ with a capacity retention of 84.6%. However, the cycle life of MCMB in 0.2-DGM was limited to only 128 cycles and failed due to an internal short circuit (Fig. 1h). Thus, an in-depth exploration is needed to identify the reasons for the performance differences mentioned above.

### Electrochemical reaction mechanism and interface characterization

**Fig. 2:**
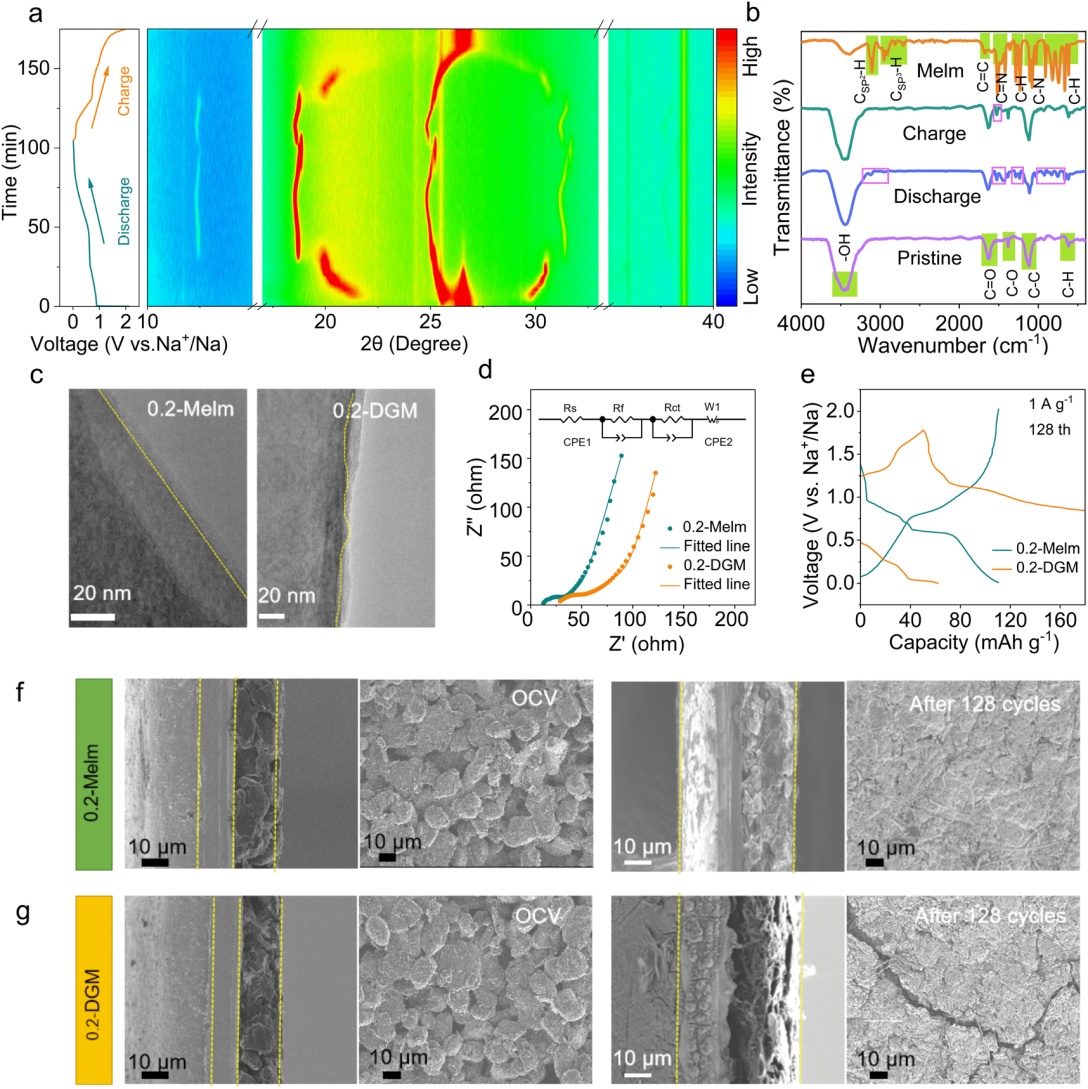
(a) Discharge/charge curves and corresponding *in situ* XRD patterns of the MCMB anode in 0.2-Melm. (b) FTIR spectra of Melm and the MCMB anode at different states. (c) HRTEM images of MCMB in 0.2-Melm and in 0.2-DGM after discharge. (d) EIS spectra of MCMB anodes in 0.2-Melm and in 0.2-DGM after 3 cycles. (e) Discharge/charge curves of MCMB anodes in 0.2-Melm and 0.2-DGM at 1 A g^−1^ in the 128th cycle. (f and g) SEM images of MCMB anodes in 0.2-Melm or in 0.2-DGM at open circuit voltage (OCV) in the 128th cycle.

To gain insight into the sodium storage mechanism of MCMB in 0.2-Melm electrolyte, *in situ* X-ray diffraction (XRD) measurements were conducted. Fig. 2a shows that the entire intercalation and extraction process is a staging behavior, similar to the intercalation of solvated sodium ions in ether.^25,27^ The peak at 26° in the initial state corresponds to the (002) plane of MCMB.^28^ During the discharge process, (002) slowly shifts to 25°, and two new peaks (at ∼20° and 30°) gradually appear, representing different stages of intercalation reactions. As the discharging occurs, the ongoing displacement suggests that the insertion of solvated Na^+^ at this stage alters the interlayer spacing.^29^ Upon discharge to 0.67 V, MCMB shows new peaks at 12.5° and 18.6°, indicative of a new stage of solvated Na^+^-intercalated graphite. However, unlike at high potential, there is almost no change in interlayer spacing, which is possibly attributable to different solvation forms at this potential.^27^ During the charging process, the peaks at ∼26° and ∼30° are not fully recovered, suggesting the presence of residual solvated Na^+^ between the layers.^25^ To determine whether it is co-embedded in a solvated form, *ex situ* Fourier transform infrared (FTIR) spectra were obtained (Fig. 2b). To avoid the influence of Melm adsorption, electrodes after cycling were cleaned multiple times with ethanol. When discharged to 0.01 V, the appearance of the C=N peak of Melm at 1500 cm^−1^ indicates the embedding of Na^+^ in a solvated form.^30^ Charging the electrode to 2 V reveals a weak peak of C=N, confirming the presence of residual solvated sodium ions between the layers, aligning with the XRD pattern. The above mechanisms played a positive role, as evidenced by the superior rate performance compared to ether (Fig. S11†), with no cracks in the MCMB particles after 100 cycles (Fig. S12†). Therefore, the residual solvated Na^+^ proves beneficial to reaction kinetics and interlayer interactions.

The investigation of interface properties remains crucial in understanding and optimizing electrochemical performance. High-resolution transmission electron microscopy (HRTEM) images reveal a more uniform solid-electrolyte interphase (SEI) film in 0.2-Melm compared to 0.2-DGM (Fig. 2c). Through X-ray photoelectron spectroscopy (XPS) analysis, it was found that the SEI layer is enriched with sodium nitride inorganic content (401.5 eV) (Fig. S13†),^31,32^ enhancing the mechanical strength and ionic conductivity of the interphase layer. Electrochemical impedance spectroscopy (EIS) of the cells after 3 cycles was performed and simulated.^33,34^ Results indicate that solution resistance (*R*_s_), interface film resistance (*R*_f_), and charge transfer resistance (*R*_ct_) are all smaller when using Melm compared to the ether electrolyte (Fig. 2d and Table S2†). This behavior can be attributed to the solvent properties, the inorganic-rich characteristics of the SEI film, and the co-solvent intercalation mechanism of Melm. The demise of ether electrolyte batteries is elucidated in Fig. 1h. Examining the discharge/charge curves of the MCMB anode over 128 cycles, it is evident that in 0.2-Melm, the curves remain normal, while in 0.2-DGM, the battery fails to charge to the set voltage (Fig. 2e). This may be due to the uneven SEI layer in 0.2-DGM, causing oxidized carbon atoms in the charged state to dissolve into the electrolyte, migrate to the sodium metal anode, and undergo redox reactions.^35^ This is supported by NMR results (Fig. S14 and Table S3†), with the C content in 0.2-DGM significantly increasing after 128 cycles compared with 0.2-DGM before cycling, indicating that carbon atoms can be dissolved into 0.2-DGM. Then in simpler terms, an internal short circuit occurs due to internal redox reactions. Furthermore, scanning electron microscope (SEM) images, along with side-view SEM images of MCMB under OCV and after 128 cycles in different electrolytes, were collected (Fig. 2f and g). Before cycling, the particles are tightly bound to the current collector, but the difference is obvious after 128 cycles. In 0.2-Melm, electrode materials remain closely bound to the current collector, presenting a smooth and flat surface. Conversely, in 0.2-DGM, cracks become apparent on the electrode surface, and the materials detach from the current collector. This may be caused by uneven stress between the active material and the current collector due to the uneven SEI.^36^

### Electrochemical performances of MCMB//NTP/C full cells

**Fig. 3:**
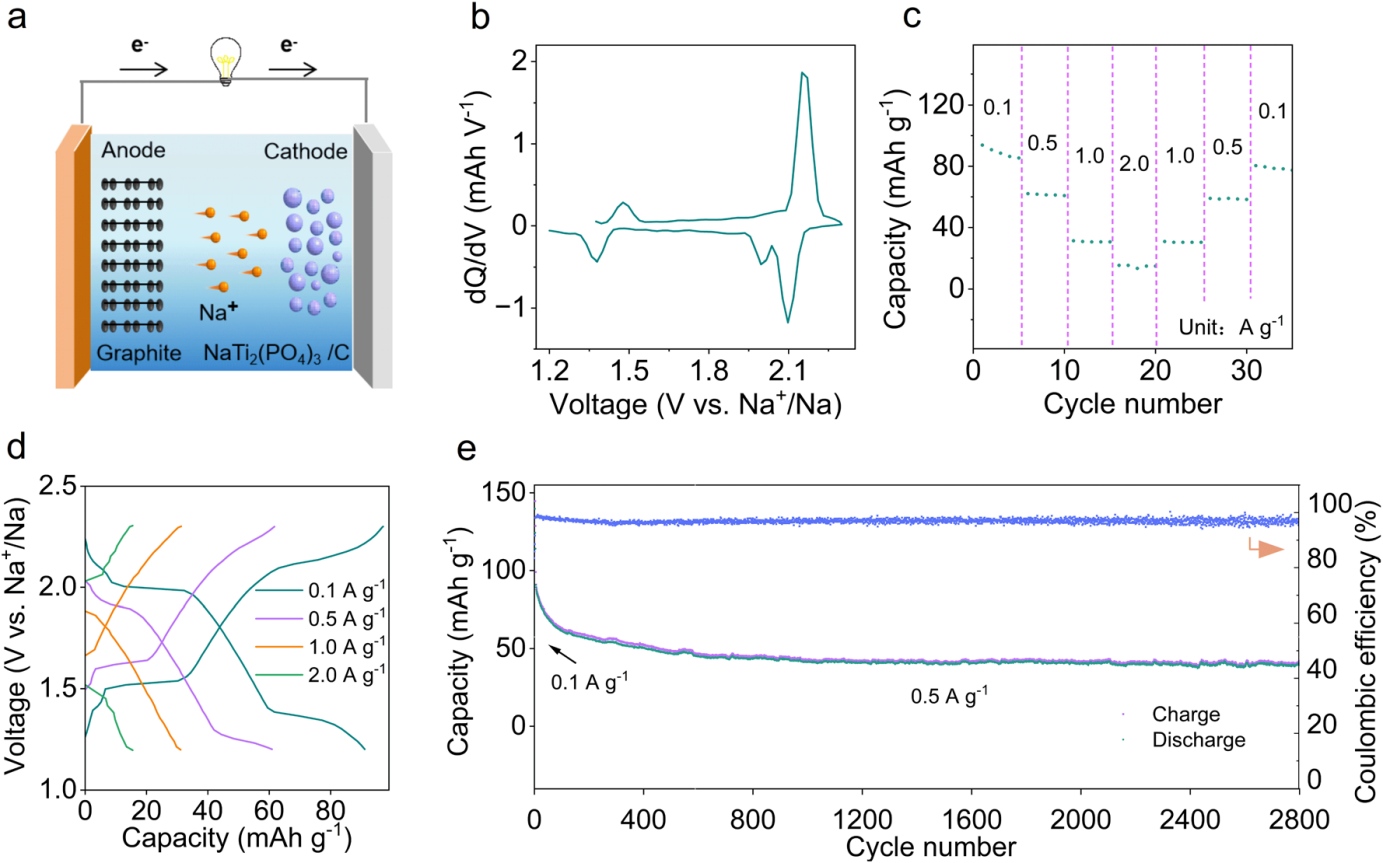
(a) Schematic diagram of the discharge process. (b) d*Q*/d*V* curves for the first cycle. (c) Rate performances. (d) Charge/discharge curves at different current densities. (e) Cycling performance at 0.5 A g^−1^.

Limited by the decomposition voltage of the electrolyte (Fig. S15†), it is unsuitable for a high voltage cathode, so NaTi_2_(PO_4_)_3_ (NTP) was chosen as the cathode.^37^ The synthesized NTP/C exhibits a size of 5–20 μm and a capacity of 130 mA h g^−1^ at a current density of 0.1 A g^−1^ (Fig. S16†). Fig. 3a illustrates the testing of full cells within 1.2–2.3 V, with the N : P ratio (capacity ratio of anode to cathode) controlled at ∼1.2. Before assembling the full cells, both the anode and cathode undergo activation for 3 cycles. To gain a clearer understanding of the reaction, the capacity *versus* voltage (d*Q*/d*V*) was integrated.^38^ The redox peaks at 1.35/1.4 V and 2.0–2.1/2.15 V correspond to the co-intercalation/de-intercalation of solvated Na^+^ in graphite (Fig. 3b). In terms of rate performance (Fig. 3c), the MCMB//NTP/C cell displays a capacity of 90.9 mA h g^−1^ at 0.1 A g^−1^, 61.5 mA h g^−1^ at 0.5 A g^−1^, 39.4 mA h g^−1^ at 1 A g^−1^, and 15.4 mA h g^−1^ at 2 A g^−1^. The capacity can be restored when the current density returns to 0.1 A g^−1^, and all capacities are obtained based on the mass of MCMB materials. With increasing current, the polarization voltage gradually rises (Fig. 3d), and the MCMB//NTP/C cell shows a capacity retention of 46.7% after 2800 cycles at 0.5 A g^−1^ (Fig. 3e). The stable cycling performance is related to the formation of a robust SEI film and the co-solvent intercalation mechanism in 0.2 Melm.

### Properties of Melm derivatives as electrolytes

**Fig. 4:**
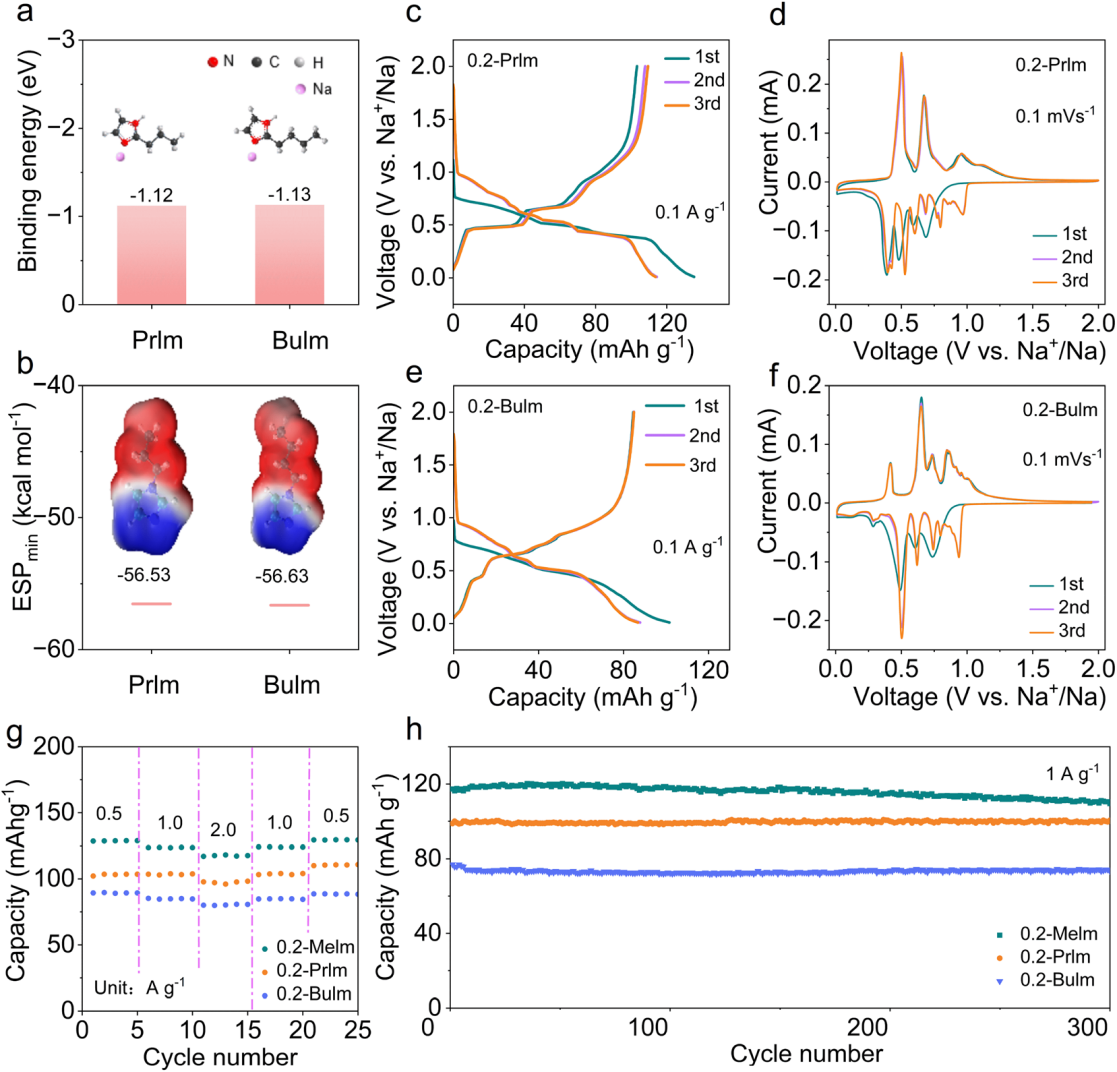
(a) The binding energy of Na^+^ and Prlm/Bulm solvents. (b) Calculated minimum electrostatic potentials (ESP_min_) of Prlm and Bulm solvents. (c) Galvanostatic voltage profiles of MCMB anode in 0.2-Prlm. (d) CV curves of MCMB anode in 0.2-Prlm. (e) Galvanostatic voltage profiles of MCMB anode in 0.2-Bulm. (f) CV curves of MCMB anode in 0.2-Bulm. (g) Rate performances and (h) long-term cycling performances of MCMB anodes in different imidazole-based electrolytes (Melm, Prlm, and Bulm).

Motivated by the favorable performance of the 0.2 Melm electrolyte, we explored the potential of imidazole derivatives, specifically Prlm and Bulm, as electrolyte solvents in SIBs due to their similar molecular structures. It is necessary to investigate whether there is a strong binding energy between Na^+^ and solvent molecules. The binding energies and the ESP_min_ of Prlm/Bulm and Na^+^ were calculated by density functional theory (DFT). The binding energies for Prlm and Na^+^ and Bulm and Na^+^ are −1.12 and −1.13 eV, respectively (Fig. 4a). The values for the ESP_min_ of Prlm and Bulm are −56.53 and −56.63 kcal mol^−1^ (Fig. 4b). The high binding energy and low ESP_min_ indicate that both Prlm and Bulm have strong binding energy with Na^+^.^39,40^ Then, the electrochemical performances of MCMB anodes in 0.2-Prlm and 0.2-Bulm were measured. At a current density of 0.1 A g^−1^, the capacity of MCMB anodes in 0.2-Prlm (116 mA h g^−1^) surpassed that in 0.2-Bulm (90 mA h g^−1^), which is potentially attributable to solvation forms and steric effects.^41^ The ICEs of MCMB anodes in 0.2-Prlm and 0.2-Bulm are 76.3% and 83.3% (Fig. 4c and e), which may be related to the different thickness of the SEI layer formed.^42,43^ The CV curves of 0.2-Prlm and 0.2-Bulm were similar to those of 0.2-Melm (Fig. 4d and f), signifying the occurrence of intercalation and deintercalation reactions of solvated sodium ions. The dissimilarity in the first cycle can be attributed to SEI formation, while subsequent cycles exhibit consistent reversibility. Different redox peaks in CV curves represent the reaction of solvated Na^+^ of different stages, that is, the formation of different GICs.^44^ Melm derivatives (0.2-Melm, 0.2-Prlm, and 0.2-Bulm) exhibited good rate performance with little capacity fading as the current increases (Fig. 4g). The long-term cycle performance of MCMB anodes in Melm derivatives as electrolytes remained stable without short circuits after 300 cycles at a current density of 1 A g^−1^ (Fig. 4h). Therefore, imidazole-based electrolytes have promising potential for applications in medium-voltage sodium-ion batteries.

## Conclusion

In summary, 0.2-Melm successfully improves the cycling performances of graphite anodes in SIBs. The Na^+^-Melm co-intercalation mechanism is substantiated through *in situ* XRD patterns and *ex situ* FTIR spectra. Notably, the residual solvated Na^+^, persisting after the initial intercalation, assumes a crucial role in the system. This residual Na^+^ not only expedites the reaction kinetics of subsequent ion insertion but also acts as a protective agent for the graphite layers, mitigating the risk of peeling off. The comparative analysis reveals that in 0.2-Melm, a more uniform SEI layer with superior ionic conductivity is established compared to 0.2-DGM, contributing to the long-term cycling performance. Based on this electrolyte, MBMC//Na batteries exhibit a discharge capacity of 150.5 mA h g^−1^ and a high ICE of 87.6%. Impressively, the reversible capacity after 1800 cycles remains substantial at 98.8 mA h g^−1^, demonstrating an outstanding capacity retention of 84.6%. Furthermore, even an MBMC//NTP/C full cell demonstrates stable cycling for an extended period of 2800 cycles at 0.5 A g^−1^. In addition, imidazole derivatives Prlm and Bulm exhibit stable long cycles in graphite anodes, surpassing the performance of ether-based electrolytes. Our research introduces a new electrolyte system for sodium-ion batteries.

## Data availability

All experimental and characterization data and detailed experimental procedures are available in the published article and ESI.†

## Author contributions

Data curation, investigation, writing – original draft: W. Zhao, conceptualization: C. Wang, data curation: Z. Cheng, methodology: C. Zheng, data curation: Q. Yao, methodology, project administration, supervision, validation: J. Pan, resources: X. Ma, project administration: J. Yang.

## Conflicts of interest

There are no conflicts to declare.

## Supplementary Material

SC-015-D3SC06640A-s001
