# Supplementary material for: Revealing the Na storage behavior of graphite anodes in low-concentration imidazole-based electrolytes
Source: Chem Sci. 2024 Apr 5;15(17):6500–6. doi: 10.1039/d3sc06640a (PMC11062126; doi:10.1039/d3sc06640a)
Supplement: SC-015-D3SC06640A-s001 [file SC-015-D3SC06640A-s001.pdf]

Electronic Supplementary Material (ESI) for Chemical Science.
This journal is © The Royal Society of Chemistry 2024

Supporting Information

Revealing the Na Storage Behavior of Graphite Anode in Low-Concentration Imidazole-Based Electrolyte

Wei Zhao,[a] Chunting Wang,[a] Zhenjie Cheng,[a] Cheng Zheng,[a] Qian Yao,[a] Jun Pan,[b] Xiaojian Ma*[a] and Jian Yang[a]*

[a]Key Laboratory of Colloid and Interface Chemistry Ministry of Education School of Chemistry and Chemical Engineering, Shandong University, Jinan 250100, P. R. China

[b]School of Physical and Mathematical Sciences, Nanyang Technological University, Singapore 637371, Singapore

*Corresponding authors.

Email: jun.pan@ntu.edu.sg; maxj@sdu.edu.cn.

Content

1. Experimental Section 4

Materials...4

Materials characterization...4

Electrochemical measurements 4

Calculation methods..5

2. Supplementary Figures...6

Figure S1 6

Figure S2 7

Figure S3 8

Figure S4 8

Figure S5 9

Figure S6 9

Figure S7 10

Figure S8 10

Figure S9 11

Figure S10 11

Figure S11 12

Figure S12 12

Figure S13 13

Figure S14 13

Figure S15 14

Figure S16 14

3. Supplementary Tables...15

Table S1 15

Table S2 15

Table S3. 16

References 17

1. Experimental Section

Materials: All chemicals were purchased commercially and were not purified. All electrolytes were prepared in an Ar-filled glove box (O_2 < 0.1 ppm and H_2O < 0.1 ppm).

Materials characterization: X-ray diffraction (XRD) patterns were obtained from a diffractometer (Rigaku, SmartLab, Japan) with Cu Kα as radiation (λ = 1.5418 Å). Scanning electron microscope (SEM) images were performed on a field-emission scanning microscope (Zeiss Gemini 300, Germany). Raman spectra were characterized on a micro-Raman spectrometer (Horiba JY Lab RAM HD88, Japan) with an excitation of 532 nm. High-resolution transmission electron microscope (HRTEM) images were acquired by a transmission electron microscope (JEOL JEM-2100F). X-ray photoelectron spectra (XPS) were measured on an X-ray photoelectron spectrometer (ESCALAB 250Xi+, Thermo Fischer, USA) using Al Kα radiation. Fourier-transform infrared (FT-IR) spectra were recorded using an infrared spectrometer (Bruker Tensor II, Germany). The viscosity was tested on a viscosity meter (KEJING STAR, MSK-SFM-VT8S, China). The ionic conductivity was measured on an ionic conductivity meter (LEICI, DDSJ-308F, China). ^{13}C NMR nuclear magnetic resonance spectra were measured on an AVANCE III HD 400 spectrometer (AVANCE NEO, Brucker, Germany) and d-DMSO was used as the solvent.

Electrochemical measurements: The mesophase carbon microbeads (MCMB) anode was fabricated by mixing MCMB powder and sodium alginate in a weight ratio of 9:1 with water as solvent. $NaTi_2(PO_4)_3$/C (NTP/C) was prepared using the reported method.[1] The NTP/C cathode was made of NTP/C powder, acetylene black, and polyvinylidene fluoride mixed in a weight ratio of 8:1:1, using 1-methyl-2-pyrrolidone as the solvent. The above slurry was ball milled for 4 hours, spread on the current collector (Cu or Al), and vacuum dried at 70°C for 12 h. Then it was cut into disks with a diameter of 12 mm, using Na metal as the counter electrode of the half-cell and NTP/C as the counter electrode of the full-cell. The active mass loading of the anode electrode is 1.3 mg cm^{-2} and the N/P = 1.2:1 with pre-lithiation (where N/P is the

capacity ratio of the anode to the cathode). The CR2032-type coin cells for galvanostatic discharge/charge were assembled in an argon-filled glove box (Mikrouna, Super 1220/750/900, H_2O<1 ppm, O_2<1 ppm) with glass fiber (Whatman GF/F) as the separator and 100 μL electrolyte in each cell. Galvanostatic discharge/charge profiles were tested on a battery-testing system (Land CT2001A, China) within 0.01-2.0 V/1.2-2.3 V. Cyclic voltammetry (CV) curves were collected on an electrochemical workstation (CHI 760E, China) within 0.01-2.0 V at the scanning of 0.15 mV s^{-1}. Electrochemical impedance spectra (EIS) and linear sweep voltammetry (LSV) curves were measured on an electrochemical workstation (Autolab PGSTAT302N, Switzerland).

Calculation methods: The binding energies between Na^+ and different solvents were evaluated using density functional theory (DFT),[2] which was operated using the Gaussian 16 package based on the Becke's three-parameter hybrid method with the Lee-Yang-Parr correlation functional (B3LYP) and the 6-311*G(d,p) basis set.[3] The minimum values of electrostatic potential (ESP_{min}) were calculated at the B3LYP/6-311G*(d,p) level with Multiwfn software. The solvents-Na^+ binding energies (Eb) were evaluated as follows:

$$E_b = E_{sol\text{-}Na^+} - E_{sol} - E_{Na^+}$$

Where E_{sol}, E_{Na^+}, and $E_{sol\text{-}Na^+}$ are the energies of the solvents, Na^+, and the solvent -Na^+ complex, respectively.

2. Supplementary Figures

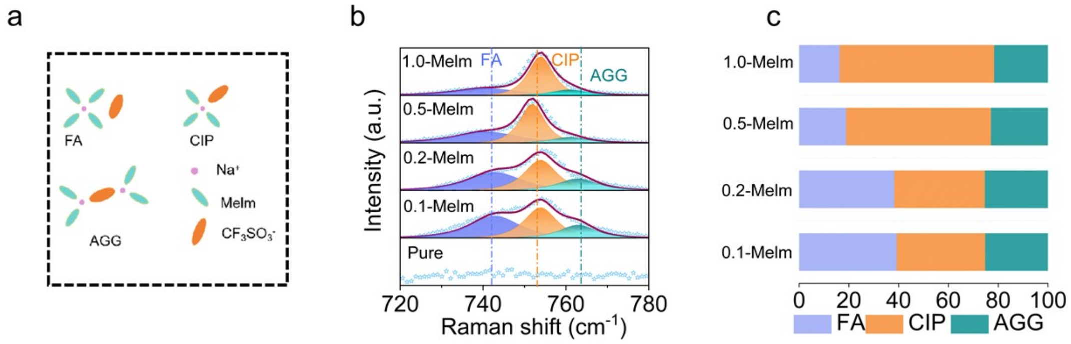

Figure S1. (a) Different combination forms of anions and cations of Na^{+}-$CF_3SO_3^{-}$. (b) Raman spectra of Melm-based electrolytes at different salt concentrations ($NaCF_3SO_3$). (c) The proportion of different combination forms of anions and cations in 0.2-Melm.

Raman spectra were detected to explore the solvation structure at different salt concentrations. The peaks located at 700-780 cm^{-1} are divided into free $CF_3SO_3^{-}$ anion (FA), contact ion pairs (CIP, $CF_3SO_3^{-}$coordinating to one Na^{+}), and aggregate (AGG, $CF_3SO_3^{-}$ coordinating to more Na^{+}).[4] Compared with 0.5-Melm and 1.0-Melm, the solvation structures of 0.1-Melm and 0.2-Melm have more FA coordination, indicating Na^{+} is encapsulated by the solvent in almost all directions. This is considered the most favorable structure and can effectively alleviate the adverse forces between Na and graphite.

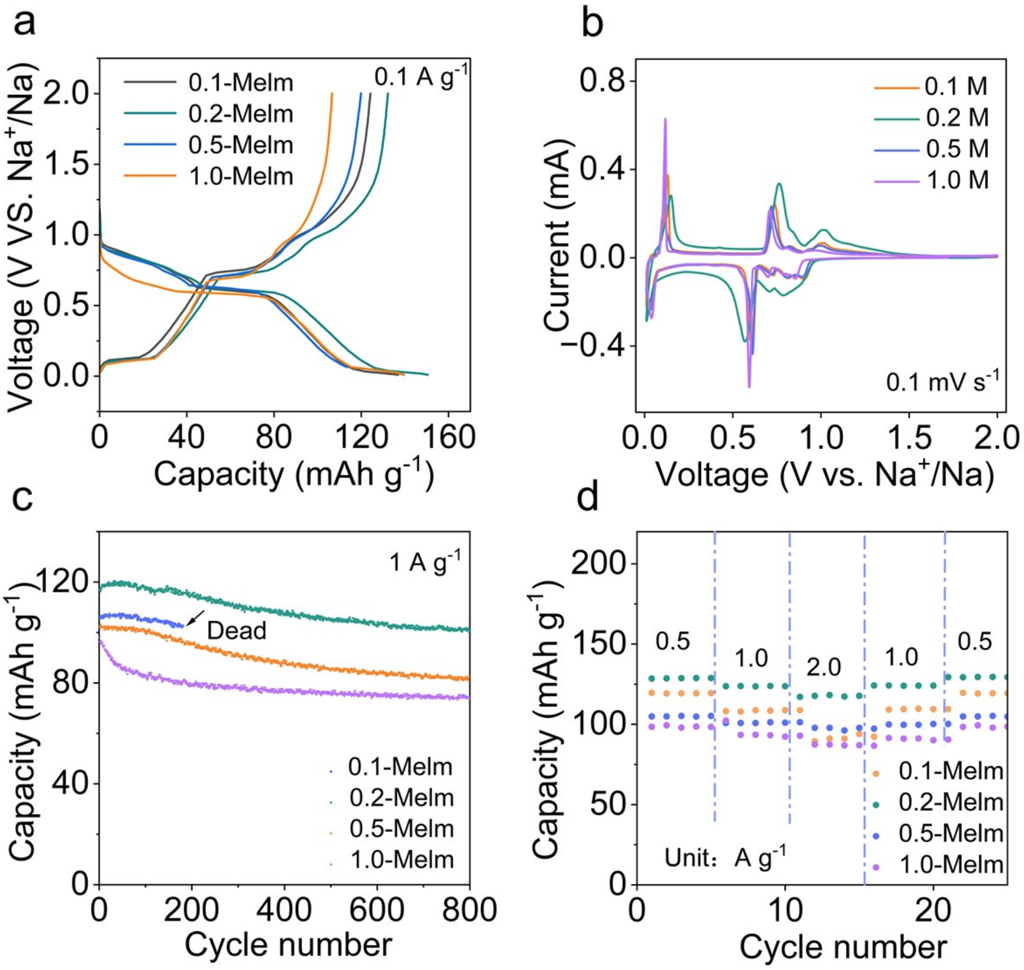

Figure S2. The electrochemical performance of MCMB anodes in Melm-based electrolytes at different concentrations. (a) Galvanostatic discharge/charge profiles. (b) CV curves. (c) Long-term cycling performances. (d) Rate performances.

Figure S2a shows that the MCMB anode has a high specific capacity of 150.5 mAh g^{-1} at 0.1 A g^{-1} with 0.2-Melm as electrolyte. Besides, Figure S2c illustrates that the MCMB anode using 0.2-Melm exhibits 100.8 mAh g^{-1} with a capacity retention of 86.4% after 800 cycles at 1 A g^{-1}. It also remains the superior capacity of 100.2 mAh g^{-1} at 2 A g^{-1} (Figure S2d). These data indicates that 0.2-Melm is the most ideal electrolyte at various salt concentrations.

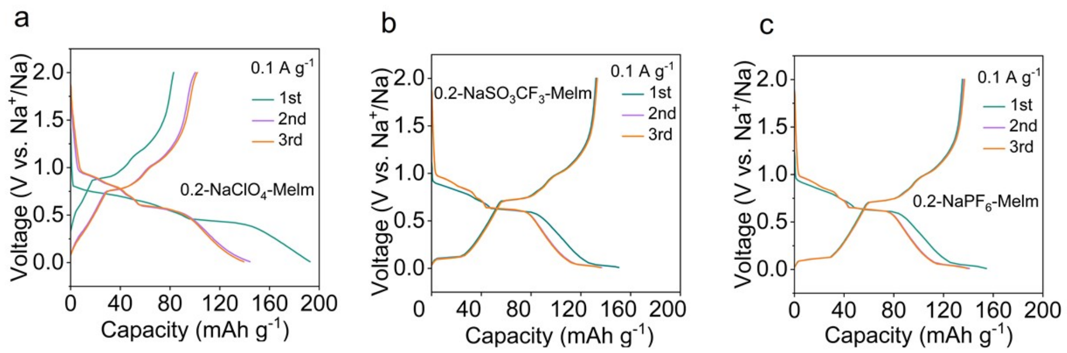

Figure S3. Galvanostatic discharge/charge profiles of MCMB anodes in different electrolytes at 0.1 A g^{-1}. (a) 0.2-$NaClO_4$-Melm, (b) 0.2-$NaCF_3SO_3$-Melm, (c) 0.2-$NaPF_6$-Melm.

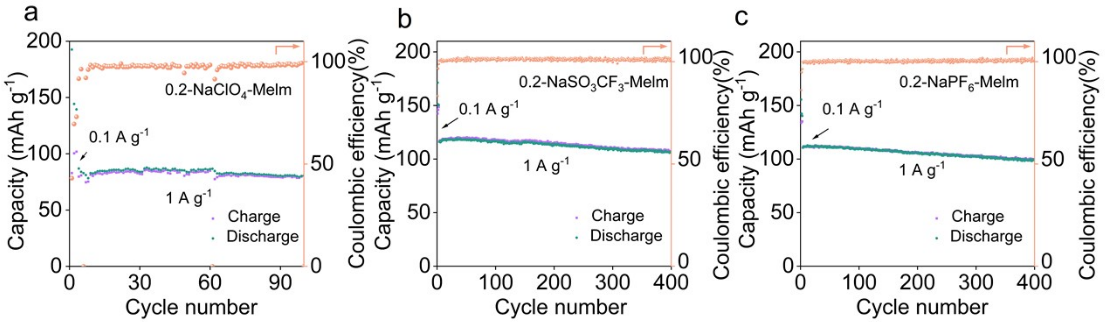

Figure S4. Long-term cycling performances of MCMB anodes in different electrolytes at 0.1 A g^{-1}. (a) 0.2-$NaClO_4$-Melm, (b) 0.2-$NaCF_3SO_3$-Melm, (c) 0.2-$NaPF_6$-Melm.

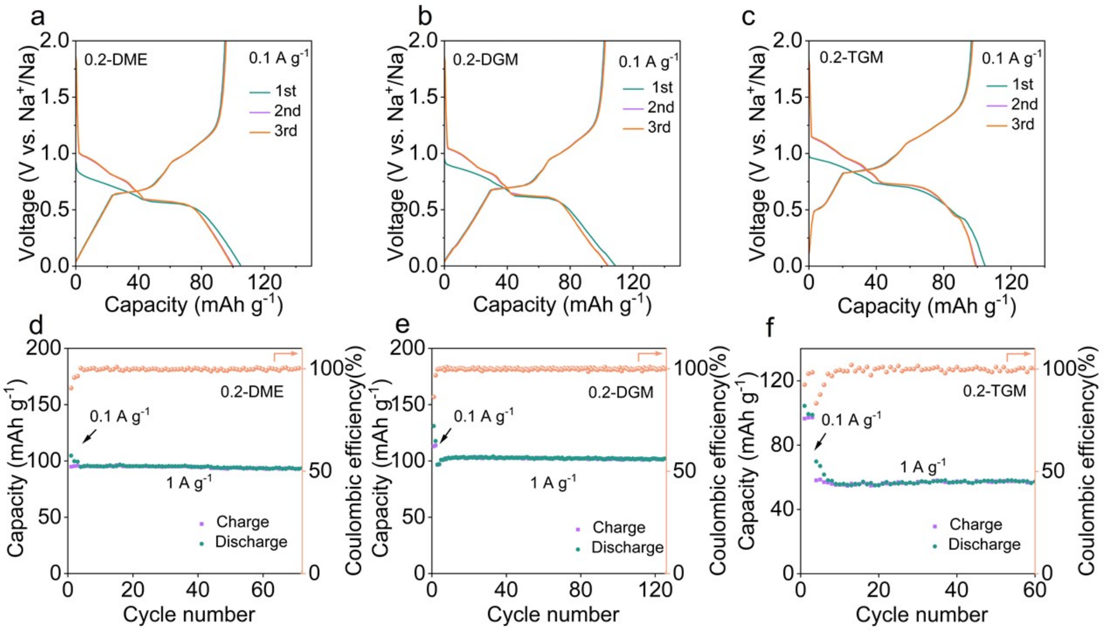

Figure S5. Electrochemical performance of MCMB anodes in ether-based electrolytes. Galvanostatic discharge/charge profiles in (a) 0.2-DME, (b) 0.2-DGM, and (c) 0.2-TGM. Long-term cycling performances in (d) 0.2-DME, (e) 0.2-DGM, and (f) 0.2-TGM.

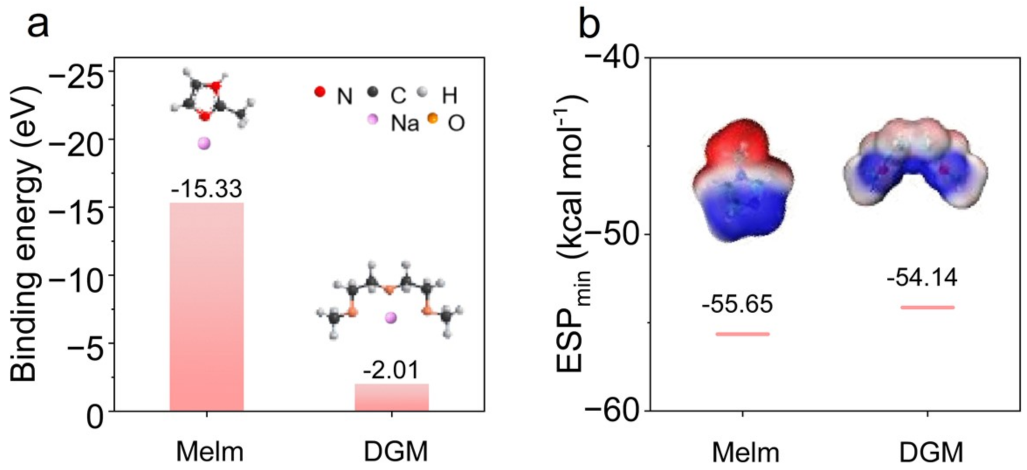

Figure S6. (a) The binding energy of Na^+ and different solvents. (b) Calculated minimum electrostatic potentials (ESP_{min}) of different solvents.

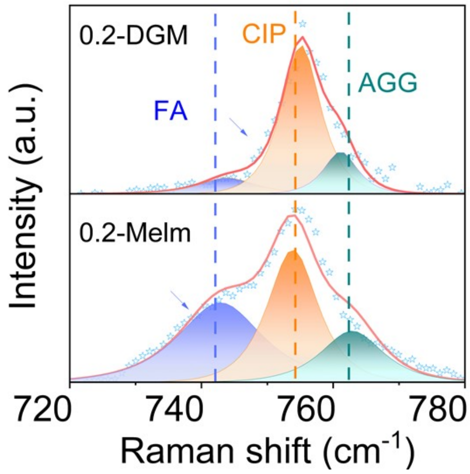

Figure S7. Different combination forms of anions and cations in 0.2-DGM and 0.2-Melm.

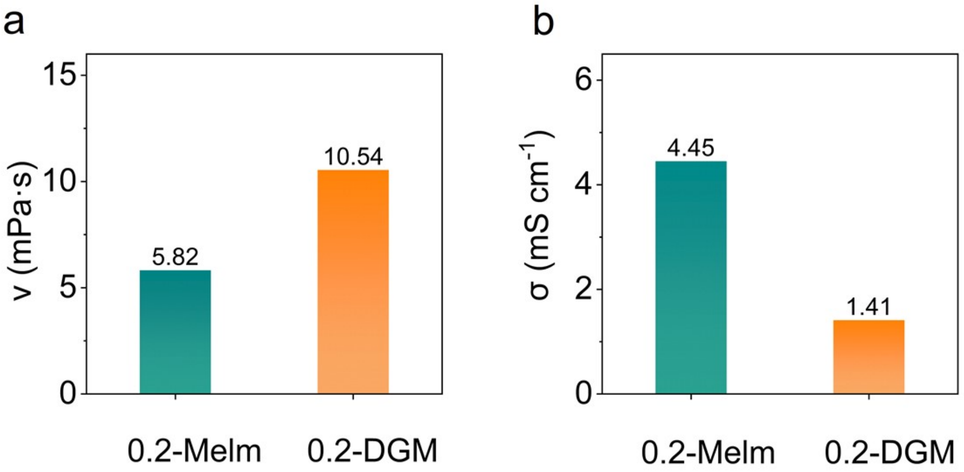

Figure S8. (a) The viscosity and (b) the ionic conductivity of 0.2-Melm and 0.2-DGM.

As calculated, the binding energy of Na^+ and Melm is -15.33 eV, which is much larger than -2.01 eV of Na^+ and DGM (Figure S6a). The ESP_{min} of Melm is lower than that of DGM (Figure S6b). Compared with 0.2-DGM, 0.2-Melm has a higher proportion of FA in Na^+-PF_6^- coordination (Figure S7). Figure S8a and Figure S8b show that 0.2-Melm exhibits lower viscosity and higher ionic conductivity than 0.2-DGM.

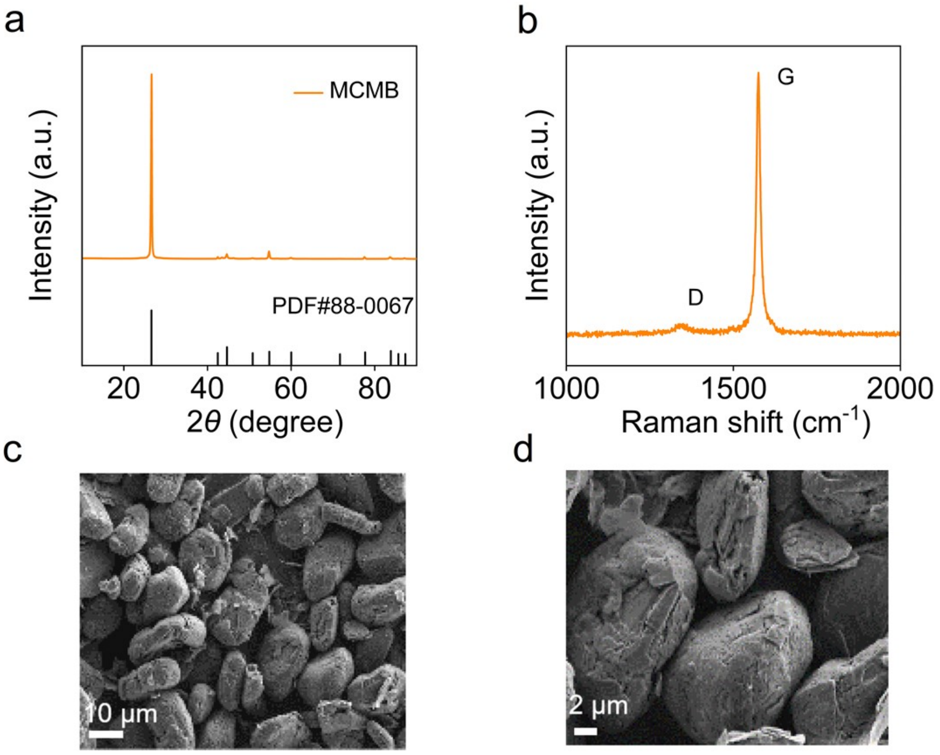

Figure S9. Properties of MCMB. (a) XRD pattern. (b) Raman spectra. (c,d) SEM images.

The strong peak located at 26.83° in XRD pattern can be directly assigned to the (002) lattice plane (Figure S9a). The Raman spectrum of MCMB shows a higher proportion of G bonds, indicating a higher degree of graphitization (Figure S9b). SEM images revealed that the size of MCMB is around 20 μm (Figure S9c and Figure S9d).

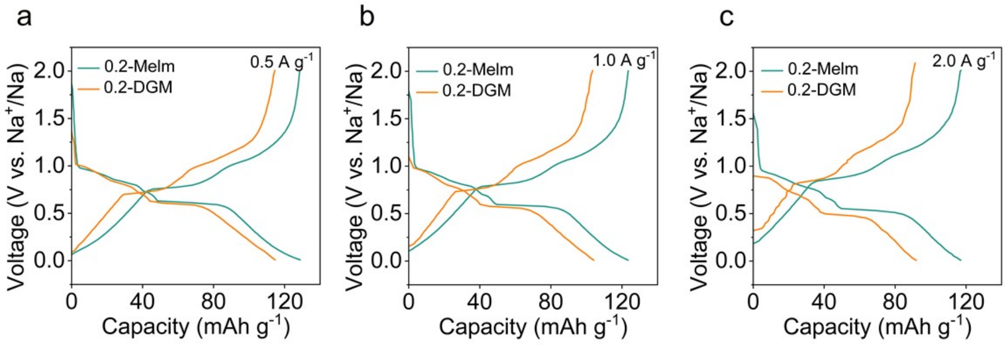

Figure S10. Galvanostatic discharge/charge profiles of MCMB anode in 0.2-Melm and 0.2-DGM at different current densities. (a) 0.5 A g^{-1}. (b) 1.0 A g^{-1}. (c) 2.0 A g^{-1}.

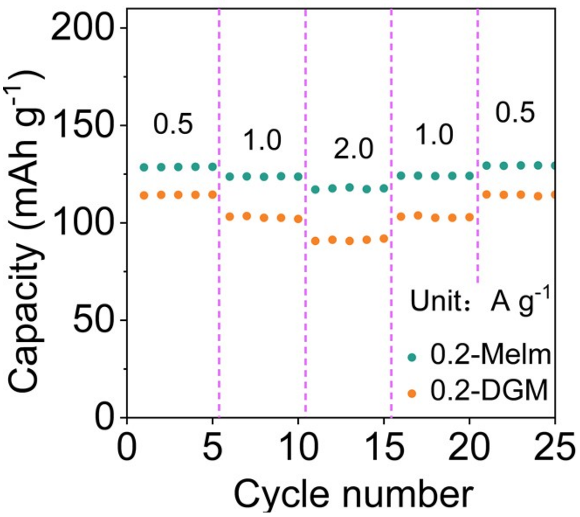

Figure S11. Rate performances of MCMB electrodes in 0.2-Melm and in 0.2-DGM.

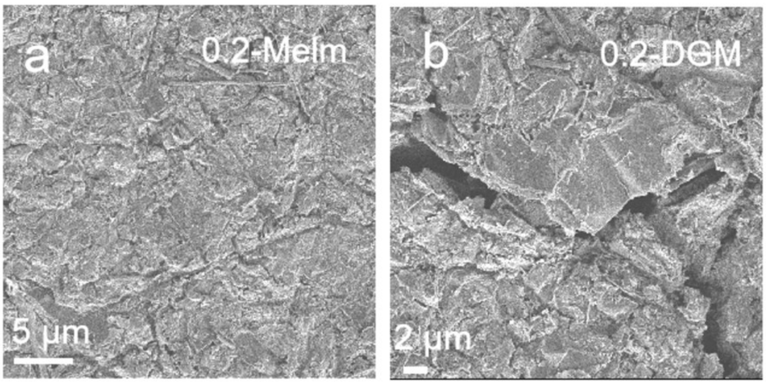

Figure S12. SEM images of MCMB anodes in 0.2-Melm (a) and in 0.2-DGM (b) after 100 cycles.

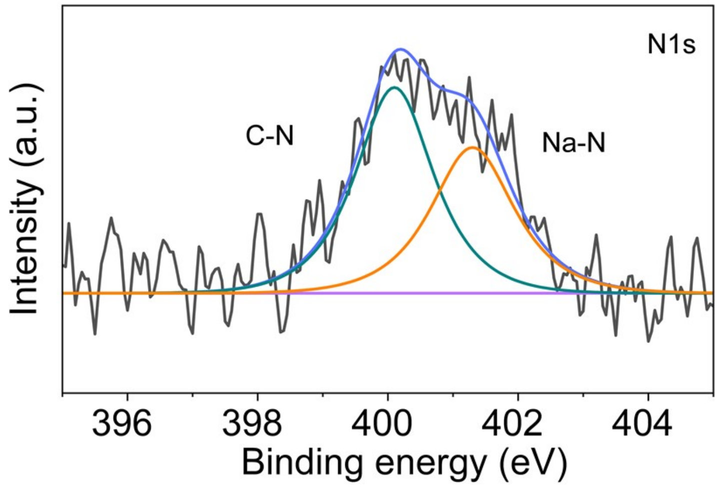

Figure S13. N 1s spectra of MCMB in 0.2-Melm after discharge.

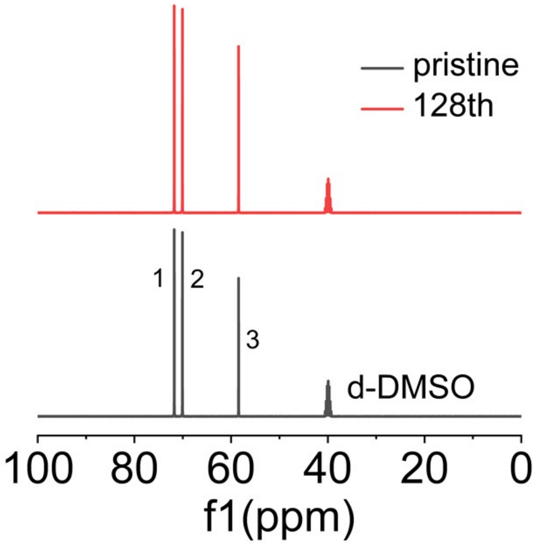

Figure S14. ^{13}C NMR of 0.2-DGM before cycling and after 128 cycles.

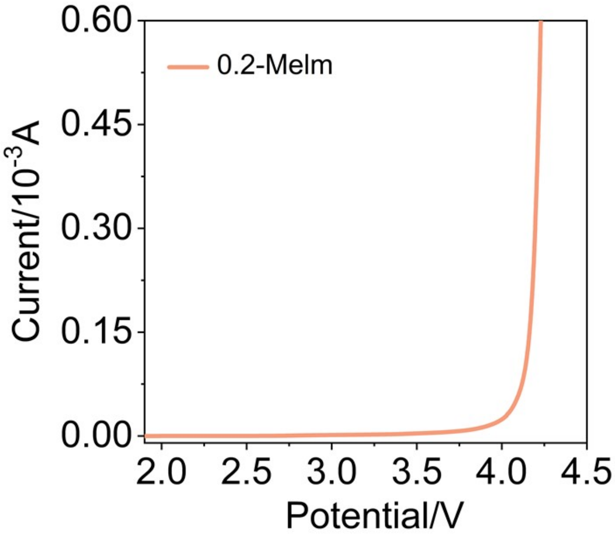

Figure S15. LSV curve of 0.2-Melm.

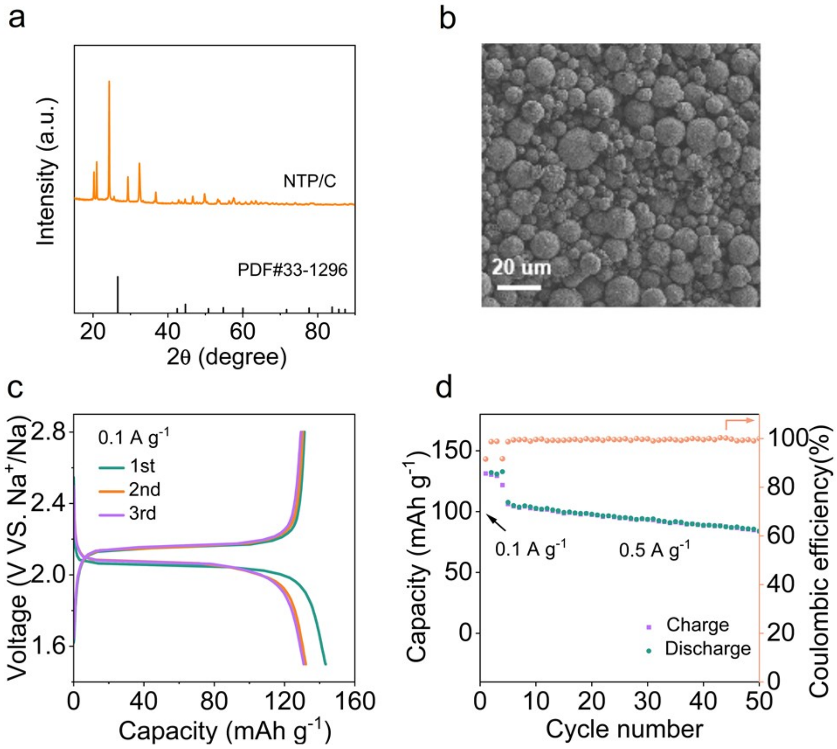

Figure S16. Properties and electrochemical performances of NTP/C. (a) XRD pattern. (b) SEM image. (c) Galvanostatic charge/discharge curves at 0.1 A g^{-1}. (d) Cycling performance at 0.5 A g^{-1}.

3. Supplementary Tables

Table S1. Compositions of the electrolytes studied in this work.

**Electrolyte**	**Salt**	**Amount of salt (mmol)**	**Solvent**	**Amount of solvent (ml)**
0.1-Melm	$NaCF_3SO_3$	0.1	Melm	1
0.2-Melm	$NaCF_3SO_3$	0.2	Melm	1
0.5-Melm	$NaCF_3SO_3$	0.5	Melm	1
1.0-Melm	$NaCF_3SO_3$	1.0	Melm	1
0.2-$NaClO_4$-Melm	$NaClO_4$	0.2	Melm	1
0.2-$NaPF_6$-Melm	$NaPF_6$	0.2	Melm	1
0.2-Prlm	$NaCF_3SO_3$	0.2	Prlm	1
0.2-Bulm	$NaCF_3SO_3$	0.2	Bulm	1
0.2-DME	$NaCF_3SO_3$	0.2	DME	1
0.2-DGM	$NaCF_3SO_3$	0.2	DGM	1
0.2-TGM	$NaCF_3SO_3$	0.2	TGM	1

Table S2. Fitting data of components in the equivalent circuit.

	**R_s (Ω)**	**R_{ct} (Ω)**	**R_f (Ω)**
0.2-Melm	8.3	7.2	6.8
0.2-DGM	11.4	17.0	16.7

Table S3. Fitting data of the NMR of 0.2-DGM before cycling and after 128 cycles.

	S_{DMSO}	S_1/ S_{DMSO}	S_2/ S_{DMSO}	S_3/ S_{DMSO}
Pristine	3521.18	0.587	0.594	0.503
128th	3305.35	0.638	0.636	0.546

References

1 W. Mao, S. Zhang, F. Cao, J. Pan, Y. Ding, C. Ma, M. Li, Z. Hou, K. Bao, and Y. Qian, *J. Alloys Compd.* 2020, **842**, 155300.

2 A. D. Becke, *J. Chem. Phys.* 1992, **97**, 9173-9177.

3 S. Grimme, S. Ehrlich, and L. Goerigk, *J. Comput. Chem.* 2011, **32**, 1456-1465.

4 Z. Tian, Y. Zou, G. Liu, Y. Wang, J. Yin, J. Ming, and H. N. Alshareef, *Adv. Sci.* 2022, **9**, 2201207.
